# Enhanced Heart Rate Prediction Model Using Damped Least-Squares Algorithm

**DOI:** 10.3390/s22249679

**Published:** 2022-12-10

**Authors:** Angela An, Mohammad Al-Fawa’reh, James Jin Kang

**Affiliations:** 1 School of Information Technology, Deakin University, Burwood, VIC 3125, Australia; 2 Computing and Security, School of Science, Edith Cowan University, Joondalup, WA 6027, Australia; 3 International College, National Taiwan University, Taipei 10617, Taiwan

**Keywords:** inference algorithm, data accuracy, data efficiency, healthcare, damped least-squares algorithm (DLSA), machine learning, neural networks, training algorithm

## Abstract

Monitoring a patient’s vital signs is considered one of the most challenging problems in telehealth systems, especially when patients reside in remote locations. Companies now use IoT devices such as wearable devices to participate in telehealth systems. However, the steady adoption of wearables can result in a significant increase in the volume of data being collected and transmitted. As these devices run on limited battery power, they can run out of power quickly due to the high processing requirements of the device for data collection and transmission. Given the importance of medical data, it is imperative that all transmitted data adhere to strict integrity and availability requirements. Reducing the volume of healthcare data and the frequency of transmission can improve a device’s battery life via an inference algorithm. Furthermore, this approach creates issues for improving transmission metrics related to accuracy and efficiency, which are traded-off against each other, with increasing accuracy reducing efficiency. This paper demonstrates that machine learning (ML) can be used to overcome the trade-off problem. The damped least-squares algorithm (DLSA) is used to enhance both metrics by taking fewer samples for transmission whilst maintaining accuracy. The algorithm is tested with a standard heart rate dataset to compare the metrics. The results showed that the DLSA provides the best performance, with an efficiency of 3.33 times for reduced sample data size and an accuracy of 95.6%, with similar accuracies observed in seven different sampling cases adopted for testing that demonstrate improved efficiency. This proposed method significantly improve both metrics using ML without sacrificing one metric over the other compared to existing methods with high efficiency.

## 1. Introduction

Healthcare data processing has become a crucial area for overcoming issues related to personal health applications. This includes aspects of battery power constraints and computational power problems involving wearables and sensor devices. This paper forms part of ongoing and continuous research [1,2] to enhance data transmission metrics, including those of accuracy and efficiency. When data are transmitted from sensors to a server in a cloud, health service providers can access and retrieve information from the server. Patients and users of healthcare services are often required to wear sensor devices such as wearables and fitness tracking devices, which capture health data and vitals, including heart rates, respiration rates, blood pressure and skin temperature. When these devices transmit collected data to a smart device, such as a smartphone, and then transfer on to a network, this process of radio transmission consumes a large amount of battery power. Thus, it is important to reduce both data size and the frequency of data transmission in order to conserve battery power. This can be achieved by improving data processing through inferencing sampling processes, increasing the data’s accuracy at the destination and the efficiency at sending devices. Previously, problems have been encountered when seeking to improve both metrics, where there has been a trade-off between metrics. Accordingly, when accuracy is increased by taking more samples, this decreases efficiency, which is measured by the number of samples taken. This paper proposes resolving this problem by using an ML training algorithm, which is shown to be achievable, as demonstrated with the DLSA. A thorough investigation has been conducted to investigate and find if a similar approach has been previously conducted, with Section 2 providing a detailed review of ML techniques and data inference algorithms and mechanisms. Kang et al. [3,4,5,6,7] focused on improving the trade-off issue between data accuracy and efficiency using a multilayer inference algorithm, which has shown significant improvement with minimal sacrifices to accuracy or efficiency. The following is a concise summary of the contributions of this paper:

- To the best of our knowledge, this paper is the first to overcome the issue of trade off between the accuracy and efficiency of telehealth systems using ML methods.
- This paper proposes an end-to-end telehealth system for the monitoring of heart rate signs in healthcare systems using a DLSA.

The rest of this paper is organized as follows: Section 2 discusses related studies that are solely focused on heart rate prediction and heart disease classification. Section 3 describes the proposed methods of telehealth system in detail. Section 4 presents the experimental details and discussion. Section 5 presents the conclusions and further directions to improve the research.

## 2. Literature Review

Many systems have been created to satisfy the requirements of developing telehealth services, and most of these have applied ML methods. This paper only focuses on heart rate prediction and heart disease classification.

Bashar et al. [8] developed a new method using a multimodal ML approach (MMLA) to predict the heart ratio using wearable sensors, mainly by applying an approach that focuses on photoplethysmography (PPG). This method consists of data collection, training, and testing for the model. The main problem associated with this step is that unusual activities, such as physical exercise, affect accuracy, and the authors subsequently used K-means clustering to remove noisy data. The final step in this method is to use random forest regression to train and predict HR. Experiments showed that the lowest mean absolute error obtained in the research was 1.11 beats per minute (BPM).

Jeyaganesan et al. [9] used data visualization techniques and ML algorithms, including decision tree (DT), logistic regression (LR), random forest (RF), and naive Bayes (NB), to predict heart disease. The authors collected their dataset, extracted 14 features, and then applied them to preprocessing methods. Finally, they trained and tested the models. The experimental results showed that RF achieved the highest precision (93.7%), greater than LR (85.7%), while DT and NB achieved precision levels of 84.5% and 83.7%, respectively.

Usman et al. [10] developed an estimation system for heart rate using speech signals in conjunction with ML algorithms. Furthermore, they applied task regression and classification algorithms, with a binary classification of heart rate as “normal” or “abnormal”, achieving 100% accuracy. At the same time, the regression method was used for the Mel frequency spectrum coefficients, representing speech features in the spectral domain and the temporal variation in the spectral features. They evaluated their model by comparing the estimated heart rate with actual measurements using a conventional medical device to record speech. They obtained an estimation accuracy of close to 94% between estimated and actual-measured heart rate values.

Ballinger et al. [11] used semi-supervised sequence learning and heuristic pretraining to detect multiple medical conditions, including diabetes, high cholesterol, high blood pressure, and sleep apnea. They collected the data from wearables such as the Fitbit, Apple Watch, or Android Wear for 57,675 person-weeks. Then, they compared the proposed model with hand-engineered biomarkers from the medical literature. Their experiment showed that the proposed method outperforms hand-engineered methods.

Chang et al. [12] constructed an AI application, a random forest classifier for heart disease problems, and the model reached an accuracy rate of 83% over the training data.

Oyeleye et al. [13] explored different data-driven models, including autoregressive-integrated moving average (ARIMA), linear regression, support vector regression (SVR), K-nearest neighbor (KNN), DT, and RF, to analyze accelerometer data to make future HR predictions from accelerometer univariant HR time-series data from healthy people using a modern dataset. The performances of these models were evaluated under different scenarios. The experimental results demonstrated the effectiveness of using an ARIMA model with walk-forward validation and linear regression for predicting heart rate under all durations and other models for durations longer than 1 min. The results of this study show that employing these methods can be used to predict future HR more accurately using accelerometers.

Li et al.’s [14] research focused on how well specific emotions experienced by pregnant women may be predicted using data related to heart rate as markers of autonomic nervous system function using ML techniques.

Alharbi et al. [15] created a real-time heart rate prediction system to help patients and medical professionals avoid heart rate risk in real-time. The suggested system is divided into offline and online phases. In the offline phase, the model was constructed using several forecasting methodologies to reduce the root mean square error. An extracted heart rate time-series dataset was obtained from Medical Information Mart for Intensive Care (MIMIC-II). Recurrent neural networks (RNN), long short-term memories (LSTM), gated recurrent units (GRU), and bidirectional long short-term memories, were all applied to heart rate time series (BI-LSTM). A three-layer GRU was then used to predict heart rate five minutes in advance.

Using wearable technology, Staffing et al. [16] collected data over ten days from twelve volunteers with varied characteristics, including age, sex, medical history, and lifestyle preferences. After being trained and tested using heart rate data gathered from twelve participants, three forecasting models (autoregressive model, LSTM, and convolutional LSTM) were compared to see which architecture performed the best in modelling and forecasting heart rate behavior. According to their trials, the autoregressive model delivered the best results for all parameters.

A statistical technique was created by Agliari et al. [17] to speculate on potential heart diseases. They gathered information from a sample of 2829 identified patients using 24 h Holter recordings. The patient data included whether or not the patients had heart diseases. In order to provide a coarse-grained definition of heart variability in terms of 49 indicators that are widely recognized in the reference community, the authors statistically analyzed the heartbeat series linked to each patient. The authors then utilized the markers as inputs to perform a principal component analysis to determine how many markers were necessary to train a neural network to categorize patients. Lastly, they trained the network and demonstrated its excellent classification accuracy (85% correct identifications at most).

Nashif et al. [18] developed a cloud-based heart disease prediction system using the support vector machine (SVM) algorithm to identify imminent heart illness. Their proposed system is trained by employing the well-known program Weka. Their SVM accuracy rate is 97.53%, with specificity and sensitivity at 94.94% and 97.50%, respectively.

Luo et al. [19] have also presented a heart rate prediction model based on an LSTM neural network to ensure the accuracy of heart rate prediction. The results of their tests demonstrate that Adam-LSTM is a reliable method for predicting heart rate and for accurately depicting trends of heart rate variation in real life. Pathak and Valan applied a decision tree to predict heart disease with 88% accuracy using the data attributes of eight patients [20]. Furthermore, Mohamed et al. used a decision tree algorithm to reduce data volume by transforming data into a more compact form to save essential features and to provide accuracy in mobile health technology [21].

One set of researchers applied optimized stacked SVM to medical applications for the prediction of early heart failure (HF). Their results reveal that this model performs better, achieving an accuracy range between 57.85% and 91.83% [22]. Another study used a fuzzy support vector machine to diagnose coronary heart disease, with experimental results showing that compared to non-incremental learning technology, this method effectively reduces the computation time of disease diagnosis [23].

An additional study deployed a naïve Bayes classifier to skin image data for skin disease detection, revealing results that outperform other methods with accuracy values ranging from 91.2% to 94.30% [24]. Similarly, Gupta et al. used Naïve Bayes for heart disease detection through feature selection in the medical sector. Their experimental results achieved 88.16% accuracy in their test dataset [25].

Researchers have attempted to use several data mining techniques to assist medical professionals in diagnosing heart disease. KNN is one of the most common data mining techniques in classification problems. For example, some researchers have proposed a novel algorithm that combines KNN and genetic algorithms to diagnose heart disease, where the proposed method aims to improve predictive accuracy. Shouman et al. [26] have investigated whether KNN can enhance accuracy by integrating other algorithms in diagnosing heart disease. Their results indicated that applying K-NN could achieve a higher accuracy rate than a neural network could for heart disease diagnosis. Shi and He noted that ANN has been intensively used for medical image processing. According to their survey, there are over 33000 items related to medical research topics of image processing that have been obtained over the last 16 years. Most of these research topics relate to medical image segmentation, preprocessing, object recognition, and detection. None of the above-mentioned studies focused on the trade-off between accuracy and efficiency. Hence, this paper will attempt to address this research gap on this trade-off between data transmission accuracy and efficiency by focusing on the regression ML technique for heart rate prediction. Table 1 outlines a comparison between our model and other related studies.

## 3. Methodology

This paper proposes a telehealth monitoring system using IoT sensors that collects biomedical data (vital signs) and then sends them efficiently to cloud servers to undergo more data analysis. The proposed model starts with data collection, then normalizes the data and sends them to a nearby cloud server to apply data mining using a well-known deep learning model called DLSA with ANN.

A. Data Collection

The first phase of the proposed model is data collection using IoT sensors (wearables). Due to resourcing and medical limitations, we used a well-known dataset that focuses on vital signs from the University of Queensland. This dataset is open access to researchers and educators. The dataset includes a wide range of patient-monitoring information, including vital signs. There are 32 subjects and 579384 samples used in this paper, including patient heart rate data. Three medical situations are available in this dataset: low, medium, and high vital signals. The case 9 [27] trend dataset, which has 6550 heart rate data points and a 1 h 49 min duration, was selected for this project, as the dataset is relatively complete compared to the other 31 case datasets.

B. Split Method

Multiple scenarios were introduced to combine possible training cases while experimenting with the training sample. Table 2 shows the testing outcomes with different ratios of each activity and using DLSA to compare and determine the best performer in terms of prediction accuracy and efficiency. The conventional ML data division method is 70% to 80% data for training, whereas the rest is for testing. This study split the data into 30% to 90% training data to build the different models and to seek the possibility of achieving relatively high accuracy and efficiency results to reduce the data size for transmission. Furthermore, it is customary to split the data into training, validation, and testing data. The training data represents the neural network training process, while the validation dataset shows the measurements of generalization and of stopping training, as the purpose of validation is to avoid overfitting problems. The testing data function was used to test the performance of neural network algorithms, providing an independent measurement of network performance.

C. Algorithm Selection

This research involves finding suitable ML algorithms that enhance healthcare data metrics. Selecting the correct algorithm when using ML is significant, as many ML algorithms can be applied. Supervised ML classification algorithms (DT, KNN, SVM, RF, and NB) are unsuitable for this study, as they attempt to classify or label data into a discrete outcome. Unsupervised algorithms are clustering algorithms that group or cluster data in similar groups through similar characteristics. Clustering algorithms are also not suitable for this task. This study aims to use ML algorithms to forecast heart rate based on the time series, which involves a regression task to predict a continuous value. Hence, the ML regression algorithm technique has been selected for this study. However, linear regression and support vector regression algorithms deal with at least two variables (independent and dependent viable) to form a close linear relationship. Accordingly, based on past values, heart rate will be predicted according to time order with a non-linear autoregressive relationship. Therefore, these are not the correct approaches for the present task.

Regarding logistic regression, this is similar to other classification algorithms that can classify labels, and they are also not in the range of choice for the regression task. Assembly methods are robust approaches to solving complex problems with large datasets, so this study did not use the ensemble method. The supervised artificial neural network algorithm can perform regression time series data tasks, especially via its outstanding regression and numerical model, which demonstrates high prediction accuracy for large and small data, making it an alternative for this study. A neural network algorithm was selected for the experiment in the next section. Furthermore, after reviewing other works, few researchers have used ML algorithms to predict heart rate in work on mobile health through focusing on a network of wearables or sensor devices. It is worth testing the algorithm with different sampling scenarios and determining the proposed method’s feasibility and acceptability [28].

DLSA is considered a predominant method for solving non-linear least-squares problems. These minimization issues are particularly prevalent when fitting least-squares curves. The gradient descent method (GDM) and the Gauss–Newton method are two numerical minimization methods that are combined in this technique (GNM). Because the DLSA is more resilient than the GNM, it frequently finds a solution, even when it starts off quite far from the final minimum. If the initial guess is reasonably close to the ideal solution, then GNM is quicker than DLSA for well-behaved functions and is suitable for beginning parameters. The trust region technique for Gauss–Newton can also be applied to DLSA. This utilizes elements of the steepest descent method to explore the design space, identify candidate solution areas, and to identify the best solution.

DLSA used to be able to train at close to second-order speeds without having to calculate the Hessian matrix. The Hessian matrix (H) can be roughly estimated as follows when the performance function takes the form of a sum of squares (as is typical during training feedforward networks:

H = J^T^J while the gradient can be computed as g = J^T^e (1)

where e is a vector of network errors, and J is the Jacobian matrix containing the first derivatives of the network errors with respect to weights and biases. In comparison to computing the Hessian matrix, computing the Jacobian matrix can be achieved using a normal backpropagation method. In the subsequent Newton-like update, DLSA approximates the Hessian matrix using the following formula:

Xk + 1 = xk − [JTJ + μI] − 1JTe,(2)

where μ is the damping factor.

Using the approximate Hessian matrix involves applying Newton’s technique when the scalar is zero. Whenever this value is large, it transforms into a gradient descent with a small step size. In this paper, DLSA has been selected following the testing of various training algorithms in artificial neural networks (ANN) featuring a computational intelligence model that can perform time-series data classification and regression tasks. DSLA optimizes self-learning to predict the output independent of the provided input. The advantage of neural networks is that they have better performance on regression tasks. This study used heart rate datasets over a period of 1 h 49 min and that included 6312 data points. Heart rates were predicted based on a historical data pattern with a period sequence. Thus, time-series neural network algorithms are appropriate for this experiment. Due to neural networks being stacked on top of this unit, the output is very non-linear. The final layer of neural networks used for prediction tasks is typically a sigmoid function. The neural network’s advantage is that it has absolute charm with regression and statistical models [29,30,31]. ANN is the most appropriate prediction algorithm and has a high prediction accuracy compared to other ML algorithms. However, one of the main disadvantages of ANN is that it requires a lot of computational work due to its iterative features [32]. The training procedure for the DLSA with the ANN is provided in Algorithm 1, whereas the details of the ANN architecture used and its hyperparameters are shown in Table 3.
**Algorithm 1: DLSA Optimization Method for Neural Network Training**1Initialize the network with a set of random parameters: weights, biases, Levenberg parameter µ2Compute Jacobian J and the approximated Hessian JTJ and the total sum of squared error3Update the weights and the biases Using the equation: xk + 1 = xk − [JTJ + μI]−1JTe4Recompute the total sum of squared error5Is the error performance Satisfactory? 6
if yes7

Safe the training weights and biases8
else
9

increase µ and re-calculate Δ θ and repeat the process from step 2 initialize the network until the last stage10end

D. Data Evaluation and Software

The ML regression technique was deployed to perform the study. MATLAB R2020b was used to generate the DLSA. An evaluation matrix was used for the supervised regression DLSP algorithm. MSE (absolute squared error), RMSE (root mean square error), MAE (mean absolute error), and MAPE (mean absolute percentage error) are widely used for evaluating the performance of regression algorithms. In addition, the coefficient of determination, known as the R-value, is significant for the prediction of accuracy as a valuable indicator in regression tasks [33]. The MSE and R-values from the models evaluate and measure the performance of neural network algorithms. In addition, MAE and MAPE can be used to evaluate accuracy (100—MAPE value). The efficiency can be calculated as follows: (total target data points/trained data points). The experiment and analysis section describes the comparison of the seven scenarios and the best-performing scenario for the algorithm. Formulas were used to evaluate and measure the accuracy and efficiency, as below.

MSE is known as the mean squared error, where n represents the total number of data points (6312 heart rates), and *Y_i_* shows the observed values of heart rate, where *i* is a certain data point, i.e., 1, 2, 3, 4 … *n*, and ${\left(\hat{Y_{i}}\right)}^{}$ is the predicted heart rate value. The statistical parameter calculates the average squared errors of the predicted heart rates and the observed original heart rate values. The lower the error between these items, the higher the accuracy.

(3) $$MSE=\frac{1}{n}\sum_{i=1}^{n}{\left(Y_{i}-\hat{Y_{i}}\right)}^{2}$$

*MAE* is the mean absolute error value, representing the average absolute error between the predicted heart rate and the observed heart rate. This directly calculates the average of the residuals. The lower *MAE* is, the higher the accuracy will be.

(4) $$MAE=\frac{\sum_{i=1}^{n}\left|Y_{i}-\hat{Y_{i}}\right|}{n}$$

*MAPE* is the sum of the average absolute error percentage and is typical for forecasting accuracy or error measurement in time series. The *MAPE* is calculated by the percentage of the mean between the observed and predicted heart rate values. A lower *MAPE* means higher accuracy. A *MAPE* value is less than 5% is considered to be a good indicator for prediction of accuracy. A *MAPE* value over 10% or more than 25% indicates low but acceptable accuracy. If the *MAPE* is greater than 25%, it means that the accuracy is low and that the model is acceptable for prediction.

(5) $$MAPE=\frac{1}{n}\sum_{t=1}^{n}\left|\frac{Y_{i}-\hat{Y_{i}}}{Y_{i}}\right|\;\times 100\%\;$$

The R-value is the coefficient of determination that represents the fitness of the predictive model. The large R-squared value indicates that there are less errors, and the model is more fit for prediction. Therefore, the highest R-value of the model was selected for accuracy. RSS is the sum of squares of residuals, and TSS is the total sum of the squares.

(6) $$R^{2}=1-\frac{RSS}{TSS}$$

The accuracy of heart rate predictions can be calculated by 100% accuracy minus the mean absolute percentage error value in the model.

(7) Accuracy=100%−MAPE 

To save battery power in sensor devices, efficiency can be considered by reducing the volume of data. The higher the efficiency is, the lower the battery consumption in the sensor device. This can be calculated by the total amount of data points (heart rates) divided by the training data points (heart rate), where 𝑛 is the total number of heart rates (6312), and 𝑡 presents the total number of training heart rates for each scenario:

(8) $$Efficiency=\frac{n}{t}$$

## 4. Experiment and Discussion

Table 4 provides information about seven different training scenarios and the results from the DLSA. The prediction accuracy is very similar, in the range of 79%, apart from Scenario 5. Regarding the lowest MSE and MAPE values as well as the higher R-values, accuracy values, and efficiency values, Scenario 7 outperforms the other six scenarios. A detailed analysis of the scenarios is discussed in Table 4.

Table 5 shows the best network performance of the DLSA from Scenario 7, using 30% of samples (1894 heart rates) to train the algorithm, 35% (2209 heart rates) for networking performance validation, and 35% for testing (2209 heart rates). The lower MSE (0.21) for the test set indicates that DLSA was the best performer of the algorithms tested. The results for the training, validation, and testing for R-value are relatively close to 1, with the testing set result (0.9977) showing the highest value across the 7 test scenarios. The MAPE (20.83%) represents the percentage average of the total error data predicted during the test. The model has relatively high accuracy and efficiency values of 79.17% and 3.33%, respectively.

**Figure 1:**
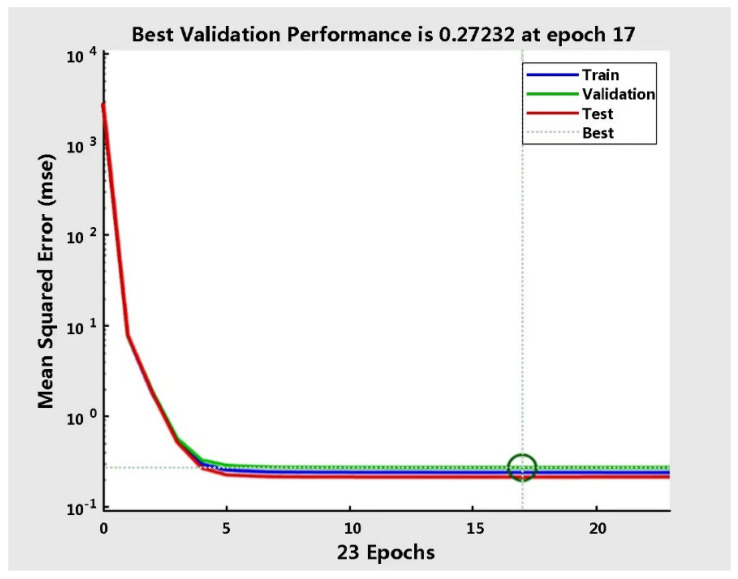
Performance of neural network at Epoch 17.

**Figure 2:**
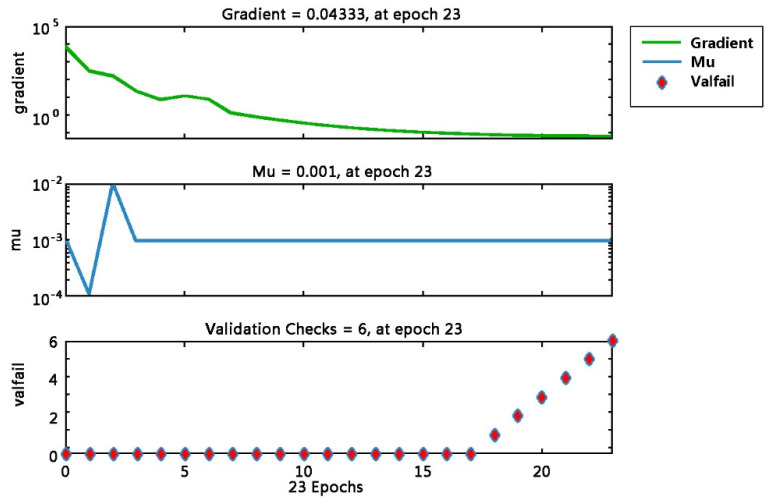
Gradient and validation checks at 6.

Figure 1 depicts the training and validation of MSE (0.27) at 17 epochs. Theoretically, the MSE is equal to 0, which means the model is perfect for prediction making. However, if the MSE value is too low, the model may have an overfitting problem. Conversely, underfitting is observed. The MES value of 0.27 shows that the model can reasonably perform prediction tasks. Figure 2 shows the results of the gradient and validation checks. The validation check is the ability of the neural network’s generalization ability to check standards. The training task will end if the error cannot be reduced after six consecutive training sessions. The gradient decrease in the cost function with the lowest error point from Epoch 17 to Epoch 23 shows no error increase. In addition, the maximum validation checks are increased from Epoch 17 to 23, reaching the default checks at 6, where the errors do not increase as the number of epochs increases.

**Figure 3:**
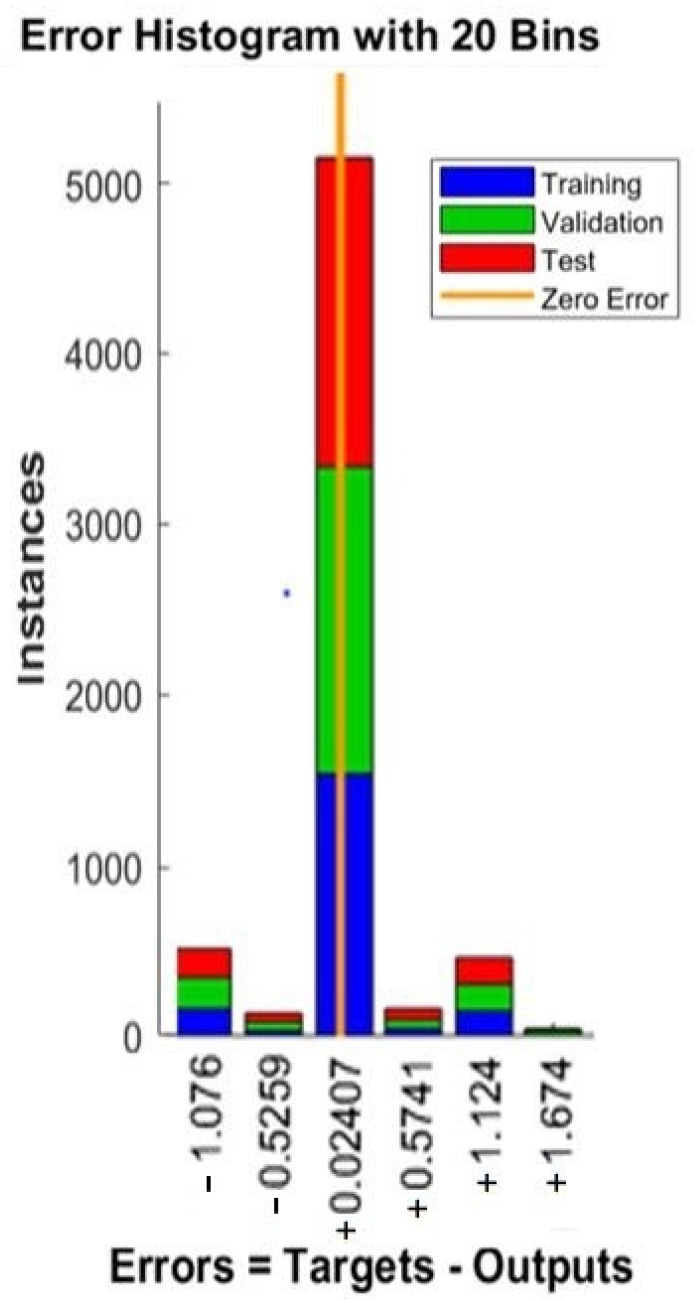
Error histograms with the lowest error values (error values close to 0).

**Figure 4:**
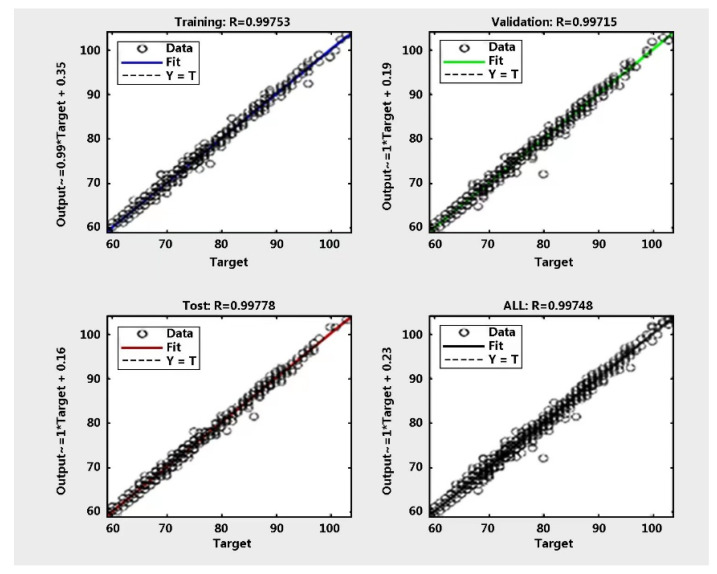
Training regression with R (test 0.997).

Figure 3 illustrates the histogram of the target-output (heart rate prediction) errors, with 20 bins between actual heart rate and predicted heart rate per second. There are 1700 training data close with to zero errors, with a heart rate prediction error of value of 0.024, which is close to zero error. Additionally, there are 1400 validation data points 1800 points. The fitting tool of the training regression algorithm results is shown in Figure 4. The R-values of the training, validation, and testing modes noted in Table 3 are all relatively close to 1, which means they are almost errorless. The model of the R-value is 0.9974.

**Figure 5:**
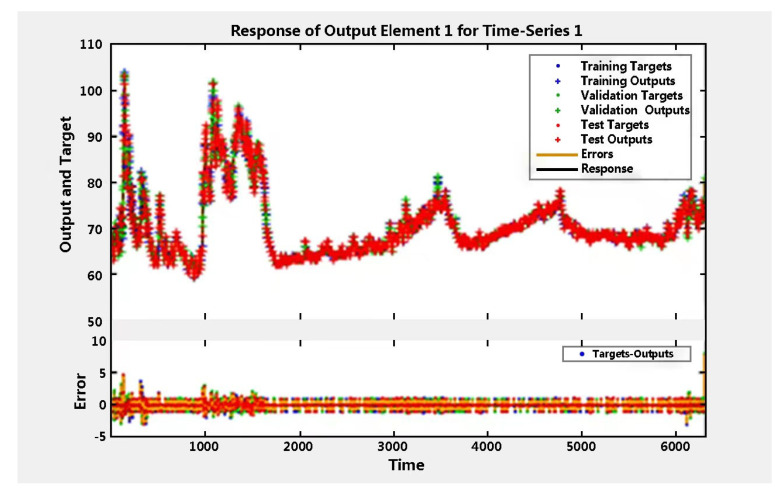
Output heart rate of time series response.

**Figure 6:**
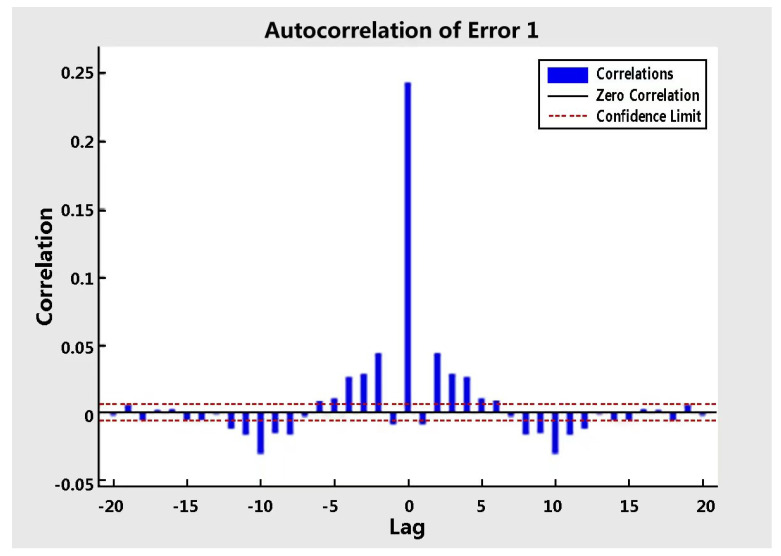
Autocorrelation of error.

Figure 5 shows that the time series reflects the seconds of an output (heart rate) as well as the errors. Figure 6 demonstrates that the autocorrelation lag of 0 equals the mean squared error at almost 0.25. This displays the same negative and positive value information symmetrically, indicating that there is high autocorrelation, and the prediction errors are related to the consecutive heart rate. In neural network training, autocorrelation plots illustrate how prediction errors are related over time. For a perfect forecasting model, the autocorrelation function should only have one non-zero value, and it should occur at zero lag. This means that the prediction errors are completely uncorrelated with each other. If there is a significant correlation in the prediction error, it should improve the prediction. In the study, with the exception of autocorrelation error one at zero lag, the correlation is approximately within the 95% confidence bound around zero, so the model appears to be adequate for accuracy. Table 5 highlights the lowest error values with MSE (0.21), MAE (1.45), and R-value (0.9977).

Over the course of seven tests, the prediction accuracy did not fluctuate in an extensive range with the different data ratios. The results showed that the accuracy was between 70 and 80% and that MAPE was around 20–21%. As mentioned before, MAPE is an important indicator for accuracy, with levels of 10–20% being acceptable, but the model considered here has lower accuracy because a MAPE value of around 5% is considered to represent high accuracy. However, efficiency can be improved significantly without compromising accuracy. This is especially the case for DLSA, where the models are pretty stable and had better convergence with all seven tests during training. This is not an expensive computation and only runs the model with 17 epochs, showing that the model is efficient and easy to train while also achieving high performance.

For comparison, another experiment was conducted using a relatively large dataset comprising heart rate data. The dataset was from a previous researcher who [4] collected 17,007 heart rates fo his previous study. We used these data to conduct the second experiment and to compare the results, as it is an open-access dataset. The data were collected from our co-author [4], and the data type is quite similar to that used in the first experiment. Table 6 shows the DLSA performance. Heart rate datasets have been used for experiments, with training data comprising 70% and 30% of the metrics including the R-value, which is the co-relation between outputs and responses, etc. MSE was deployed to measure the average square of the error between the target and output. The MSE and R-value evaluate and measure the algorithm’s performance, e.g., R (70) is the outcome of 70% of the data trained to predict 30% of the samples. This is also the case for MAE and MAPE. Table 5 depicts the results of heart rate data used for training, noted as Tr (training), V (validation), Ts (testing), Ds (dataset), and Dp (datapoints). Surprisingly, the accuracy is almost the same between the training targets (70) and (30). This indicates that ML algorithms perform very well with fewer training samples. Similarly, the MAPE values are 4.3–4.4%, which is a significant finding for a more accurate model with a large dataset and indicates that DLSA performs well with fewer training samples. This also affects other metrics, such as efficiency, which can be calculated below.

(9) $$\frac{\mathrm{Number}\;\mathrm{of}\;\mathrm{Total}\;\mathrm{Target}\;\mathrm{Samples}}{\mathrm{Number}\;\mathrm{of}\;\mathrm{Trained}\;\mathrm{datapoints}}$$

**Figure 7:**
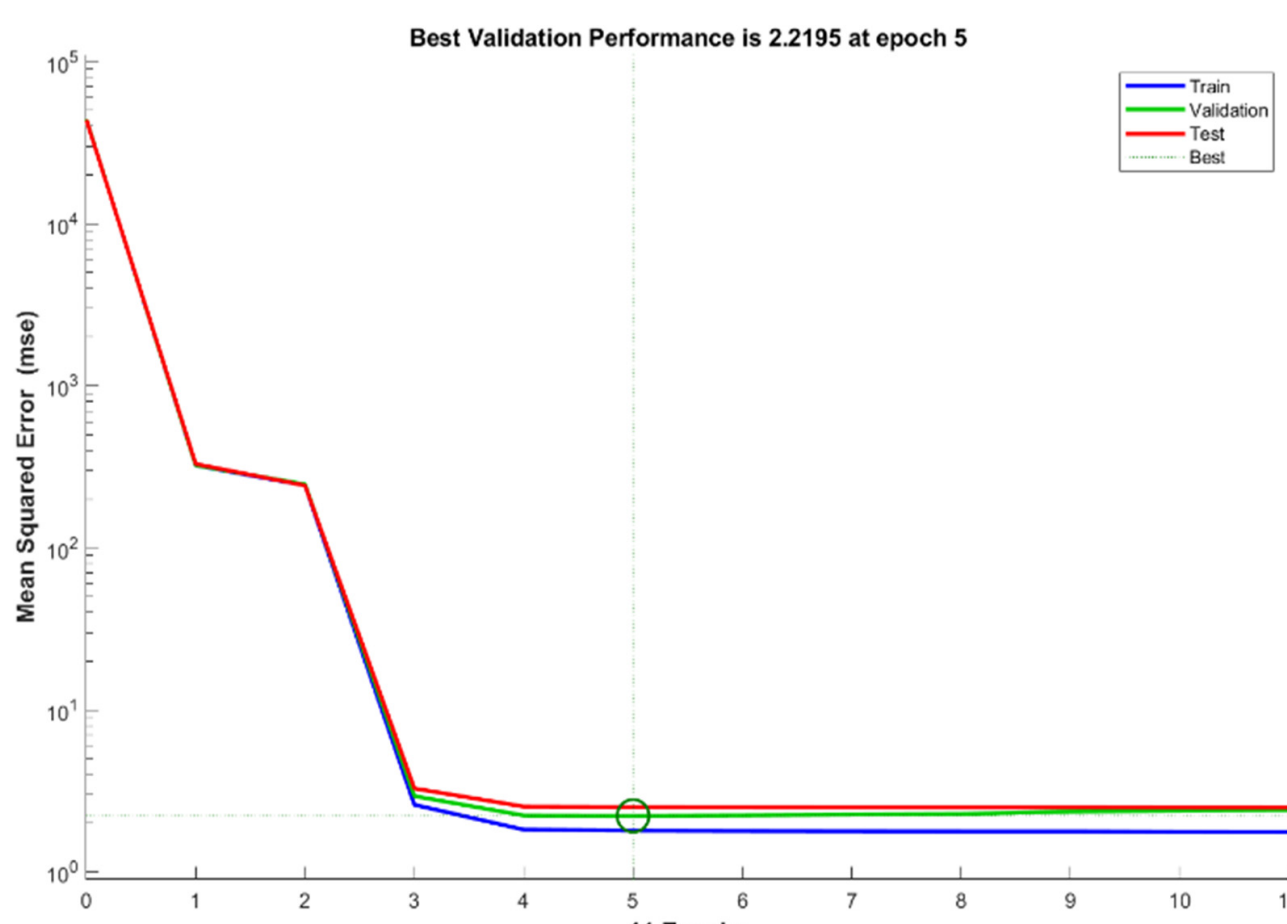
Performance of best validation at Epoch 5, which shows reduced and maintained errors.

Figure 7 shows the best performance obtained during training and validation, with an MSE (70) value at 5 epochs. No overfitting appeared during error validation, halting before Epoch 5. The error decreases from Epoch 5 and shows stable movement until Epoch 11. In the circle of Figure, it shows all three datasets (Training, validation and testing) are reached stable state that the errors are not increased with increasing of epochs. Table 6 compares the best validation performances of MSE (70) and MSE (30) to contrast the MSE outcome, which shows higher errors with fewer training data. The error is reduced significantly with 70% training data compared to 30% training data, as it takes more training to make predications with fewer errors. Comparing the outcomes of both training scenarios using 70% and 30% training data, which are almost identical except for the error marked with yellow boxed arrows, shows that the algorithm performance improves significantly without much difference in the regression value R, whilst the training size is significantly different between the 70% and 30% training data, as shown in Table 6. This means that efficiency can improve significantly with little difference in performance metrics such as accuracy. For instance, the efficiency was improved by 2.3 times whilst maintaining almost the same accuracy (a 0.1% decrease).

## 5. Conclusions and Future Work

Several ML models are available for HR forecasting. However, in this paper, DLSA was selected due to its efficiency in enhancing the metrics. This paper applied time series and non-linear autoregressive neural networks using DLSA to predict heart rates. The DLSA neural network algorithm showed stable performance in terms of the least number of errors and similar prediction accuracy, but with improved efficiency. Therefore, ML was proven to improve healthcare data metrics simultaneously compared to existing methods, which trade-off accuracy and efficiency. Thus, it is worthwhile to attempt different data ratios using the algorithm and to compare the models with high accuracy and efficiency. The metric performance may vary depending on the size of the dataset for mature ML algorithms, with more data possibly resulting in a better outcome. Whilst the accuracy does not change much, the efficiency can improve significantly. This means that it is feasible to improve efficiency by reducing the sample size and by maintaining similar accuracy levels using ML algorithms. This study continues to extend the methods with other healthcare types and sizes to determine the best metrics by optimizing training versus prediction samples.

As for future applications that can use the proposed approach, the scenarios depicted in Figure 1 can be achieved when the end-to-end network has been fully implemented from the data capture in personnel devices to presentation via a smartphone app for the user through data processing and optimization processes by network intelligence via a near-production approach. Full design and implementation works are under development using human subjects and real-time data collection. Collecting, processing, and creating a personalized health index will be challenging, as they require health data to be accumulated over a long period of time as well as ongoing monitoring for accurate prediction and training performance. Collecting real-time data and transmitting them to a centralized server in defense networks is also an area to design and develop for an end-to-end solution that should consider security and privacy aspects.

Internet of Things (IoT) devices for defense purposes can be connected with a server in a defense cloud to connect personal sensor devices and IoT or IoT-enabled devices such as sensors, personal health devices, weaponry, firearms, and communication equipment for instance. In other words, an mHealth-based sensor network used for capturing human health data can be connected to a defense IoT network to collect and transmit data to a centralized server. An example of an IoT-enabled private network is a low-powered wide-area network (LPWAN), which is typically used in the agricultural industry for crop monitoring. Wireless body-area networks (WBAN) are another example of a private network composed of IoT devices that can be integrated with an mHealth network. This refers to a collection of inter-networking devices and system architectures used for the optimal collection, classification, and delivery of health information. The journey typically starts with users/patients (capturing vital physiological signs) traversing across multiple platforms and nodes across the internet, where ML can enhance the applications and services using heart rate prediction technologies.

## Figures and Tables

**Table 1:** Performance comparison with related studies.

Model	Accuracy	Efficiency	Ref.
Random Forest	93.7%	N/A	[9]
Logistic Regression	85.7%	N/A	[9]
Naive Bayes	83.7%	N/A	[9]
Neural Network	85.0%	N/A	[17]
Adam-LSTM	88.0%	N/A	[20]
DLSA	95.6%	3.33	Our proposal

**Table 2:** Sampling scenarios of the training dataset.

Data Split Method			
Scenarios	Training Data	Validation Data	Testing Data
1	90%	5%	5%
2	80%	10%	10%
3	70%	15%	15%
4	60%	20%	20%
5	50%	25%	25%
6	40%	30%	30%
7	30%	35%	35%

**Table 3:** Proposed DLSA prediction model for setting hyperparameters.

Parameter	Value
Max number of epochs to train	1000
Performance goal	0
Max validation failures	6
Min performance gradient	1 × 10^7^
Initial mu.	0.001
Decrease factor for mu	0.1
Increase factor for mu	10
Max value for mu	1 × 10^10^
Max time to train in seconds	infinity

**Table 4:** DLSA testing data results.

Training Scenarios	MSE	R	MAE	MAPE	Accuracy	Efficiency
1	0.20	0.9981	1.43	20.51%	79.48%	1.11
2	0.23	0.9976	1.42	20.45%	79.54%	1.24
3	0.23	0.9975	1.42	20.47%	79.53%	1.42
4	0.24	0.9977	1.46	20.92%	79.08	1.66
5	0.25	0.9972	1.47	21.09%	78.91%	2
6	0.23	0.9975	1.46	20.96%	79.04%	2.5
7	0.21	0.9977	1.45	20.83%	79.17%	3.33

**Table 5:** Best performance of DLSA from Scenario 7.

Data Split Method	Training	Training Data Points	Validation	Validation Data Points	Testing	Testing Data Points
Results (%)	30%	1894	35%	2209	35%	2209
MSE	0.24		0.27		0.21	
R	0.9975		0.9971		0.9977	
MAE	1.5					
MAPE (%)	20.8					
Accuracy (%)	79.1					
Efficiency	3.3					

**Table 6:** Performance of damped least-squares scenario.

Metrics	TrDs	TrDp	VDs	VDp	TsDs	TsDp
Samples 17,007	70%	11,905	15%	2551	15%	2551
	30%	5103	35%	5952	35%	5952
MSE (70)	1.868		1.871		1.788	
MSE (30)	1.534		1.955		2.4	
R (70)	0.9979		0.9978		0.9980	
R (30)	0.9984		0.9976		0.9974	
		70% training data		30% training data		
MAE		1.119		1.223		
MAPE		4.3%		4.4		
Accuracy		95.7%		95.6%		
Efficiency		1.43		3.33		

## Data Availability

The data that support the findings of this study are openly available in [The University of Queensland Vital Signs Dataset] at [https://doi.org/10.1213/ANE.0b013e318241f7c0 (accessed on 30 October 2022)], reference number [27].

## Abbreviations

DLSA	Damped Least-Squares Algorithm
ML	Machine Learning
ANN	Artificial Neural Networks
HR	Heart Rate
MSE	Mean Squared Error
RMSE	Root Mean Square Error
MAE	Mean Absolute Error
MAPE	Mean Absolute Percentage Error
MSE	Mean Squared Error

## References

[1] An Q., Szewczyk P., Johnstone M.N., Kang J.J. Enhancement of Healthcare Data Performance Metrics using Neural Network Machine Learning Algorithms. Proceedings of the 2021 31st International Telecommunication Networks and Applications Conference (ITNAC).

[2] Kang J.J. A Military Human Performance Management System Design using Machine Learning Algorithms. Proceedings of the 2021 31st International Telecommunication Networks and Applications Conference (ITNAC).

[3] Kang J.J. A Military Mobile Network Design: mHealth, IoT and Low Power Wide Area Networks. Proceedings of the 2020 30th International Telecommunication Networks and Applications Conference (ITNAC).

[4] Kang J.J.W. (2017). An Inference System Framework for Personal Sensor Devices in Mobile Health and Internet of Things Networks. Ph.D. Thesis.

[5] Kang J.J., Yang W., Dermody G., Ghasemian M., Adibi S., Haskell-Dowland P. (2020). No Soldiers Left Behind: An IoT-Based Low-Power Military Mobile Health System Design. IEEE Access.

[6] Kang J.J., Dibaei M., Luo G., Yang W., Zheng X. A Privacy-Preserving Data Inference Framework for Internet of Health Things Networks. Proceedings of the 2020 IEEE 19th International Conference on Trust, Security and Privacy in Computing and Communications (TrustCom).

[7] Kang J.J., Sikos L.F., Choo K.K.R. (2020). Systematic Analysis of Security Implementation for Internet of Health Things in Mobile Health Networks BT—Data Science in Cybersecurity and Cyberthreat Intelligence.

[8] Bashar S.S., Miah M.S., Karim A.H.M.Z., al Mahmud M.A., Hasan Z. A Machine Learning Approach for Heart Rate Estimation from PPG Signal using Random Forest Regression Algorithm. Proceedings of the 2019 International Conference on Electrical, Computer and Communication Engineering (ECCE).

[9] Jeyaganesan J., Sathiya A., Keerthana S., Aiyer A. (2020). Diagnosis and Prediction of Heart Disease Using Machine Learning Techniques. Ilkogr. Online-Elem. Educ. Online.

[10] Usman M., Zubair M., Ahmad Z., Zaidi M., Ijyas T., Parayangat M., Wajid M., Shiblee M., Ali S.J. (2021). Heart rate detection and classification from speech spectral features using machine learning. Arch. Acoust..

[11] Ballinger B., Hsieh J., Singh A., Sohoni N., Wang J., Tison G.H., Marcus G.M., Sanchez J.M., Maguire C., Olgin J.E. Deepheart: Semi-supervised sequence learning for cardiovascular risk prediction. Proceedings of the 32nd AAAI Conf. Artif. Intell. AAAI 2018.

[12] Chang V., Bhavani V.R., Xu A.Q., Hossain M. (2022). An artificial intelligence model for heart disease detection using machine learning algorithms. Healthc. Anal..

[13] Oyeleye M., Chen T., Titarenko S., Antoniou G. (2022). A Predictive Analysis of Heart Rates Using Machine Learning Techniques. Int. J. Environ. Res. Public Health.

[14] Li X., Ono C., Warita N., Shoji T., Nakagawa T., Usukura H., Yu Z., Takahashi Y., Ichiji K., Sugita N. (2022). Heart Rate Information-Based Machine Learning Prediction of Emotions Among Pregnant Women. Front. Psychiatry.

[15] Alharbi A., Alosaimi W., Sahal R., Saleh H. (2021). Real-Time System Prediction for Heart Rate Using Deep Learning and Stream Processing Platforms. Complexity.

[16] Staffini A., Svensson T., Chung U.I., Svensson A.K. (2022). Heart rate modeling and prediction using autoregressive models and deep learning. Sensors.

[17] Agliari E., Barra A., Barra O.A., Fachechi A., Vento L.F., Moretti L. (2020). Detecting cardiac pathologies via machine learning on heart-rate variability time series and related markers. Sci. Rep..

[18] Nashif S., Raihan M.R., Islam M.R., Imam M.H. (2018). Heart Disease Detection by Using Machine Learning Algorithm Algorithms and Algorithm a Real-Time Cardiovascular Health Monitoring System. World J. Eng. Technol..

[19] Luo M., Wu K. (2020). Heart rate prediction model based on neural network. IOP Conf. Ser. Mater. Sci. Eng..

[20] Pathak A.K., Valan J.A. (2020). A Predictive Model for Heart Disease Diagnosis Using Fuzzy Logic and Decision Tree BT, inSmart Computing Paradigms: New Progresses and Challenges.

[21] Mohamed W.N.H.W., Salleh M.N.M., Omar A.H. A comparative study of Reduced Error Pruning method in decision tree algorithms. Proceedings of the 2012 IEEE International Conference on Control System, Computing and Engineering.

[22] Ali L.I., Niamat A., Golilarz N.A., Ali A., Xingzhong X. (2019). An Optimized Stacked Support Vector Machines Based Expert System for the Effective Prediction of Heart Failure. IEEE Access.

[23] Nilashi M., Ahmadi H., Manaf A.A., Rashid T.A., Samad S., Shahmoradi L., Aljojo N., Akbari E. (2020). Coronary Heart Disease Diagnosis Through Self-Organizing Map and Fuzzy Support Vector Machine with Incremental Updates. Int. J. Fuzzy Syst..

[24] Balaji V.R., Suganthi S.T., Rajadevi R., Kumar V.K., Balaji B.S., Pandiyan S. (2020). Skin disease detection and segmentation using dynamic graph cut algorithm and classification through Naive Bayes classifier. Measurement.

[25] Gupta A., Kumar L., Jain R., Nagrath P. (2019). Heart Disease Prediction Using Classification (Naive Bayes) BT. Proceedings of First International Conference on Computing, Communications, and Cyber-Security (IC4S 2019).

[26] Shouman M., Turner T., Stocker R. (2012). Applying k-Nearest Neighbour in Diagnosing Heart Disease Patients. Int. J. Inf. Educ. Technol..

[27] Liu D., Görges M., Jenkins S.A. (2012). University of Queensland vital signs dataset: Development of an accessible repository of anesthesia patient monitoring data for research. Anesth. Analg..

[28] Kulkarni P., Watwe V., Chavan T., Kulkarni S. (2021). Artificial Neural Networking for remediation of methylene blue dye using “Fuller’s earth clay”. Curr. Res. Green Sustain. Chem..

[29] Abiodun O.I., Jantan A., Omolara A.E., Dada K.V., Mohamed N.A., Arshad H. (2018). State-of-the-art in artificial neural network applications: A survey. Heliyon.

[30] Rajkumar A., Reena M.G.S. (2014). Diagnosis of Heart Disease Using Data mining Algorithm. Int. J. Comput. Sci. Inf. Technol..

[31] Chaurasia S., Vikas P. (2013). Early Prediction of Heart Diseases Using Data Mining Techniques. Caribb. J. Sci. Technol..

[32] Durairaj M., Ranjani V. (2013). Data Mining Applications in Healthcare Sector: A Study. Int. J. Sci. Technol. Res..

[33] Patel J., Tejal Upadhyay D., Patel S. (2015). Heart disease prediction using machine learning and data mining technique. Heart Dis..

